# Contribution of Second-Shell Residues to PLP-Dependent Transaminase Catalysis: A Case Study of D-Amino Acid Transaminase from *Desulfomonile tiedjei*

**DOI:** 10.3390/ijms26178536

**Published:** 2025-09-02

**Authors:** Alina K. Bakunova, Iuliia V. Rudina, Vladimir O. Popov, Ekaterina Yu. Bezsudnova

**Affiliations:** 1 Bach Institute of Biochemistry, Research Centre of Biotechnology of the Russian Academy of Sciences, Moscow 119071, Russia; alice-43@yandex.ru (I.V.R.); v.popov@fbras.ru (V.O.P.); eubez@inbi.ras.ru (E.Y.B.); 2 Department of Biology, Lomonosov Moscow State University, Moscow 119991, Russia

**Keywords:** D-amino acid transaminase, pyridoxal-5′-phosphate, cofactor, structure–function relationships, second-shell residues

## Abstract

Understanding the structure–function relationships of pyridoxal-5′-phosphate (PLP)-dependent transaminases is key to advancing pyridoxal-phosphate-dependent catalysis and engineering transaminases for industrial applications. Despite our extensive knowledge of PLP-dependent enzymatic reactions, engineering transaminase activity and stability remains challenging. Here, we present the functional characterization of a novel PLP-dependent fold type IV transaminase from *Desulfomonile tiedjei*, alongside a detailed analysis of PLP binding and holoenzyme stability. This new transaminase exhibits activity toward various D-amino acids and (*R*)-phenylethylamine. Structural modeling and site-directed mutagenesis of residues in the second shell of the PLP-binding site revealed their roles in cofactor binding and the transaminase’s catalytic efficiency. Notably, the T199Q variant demonstrated a fivefold increase in PLP affinity and improved activity under alkaline conditions. This is attributed to a newly formed hydrogen bond that stabilizes the N1-binding region of PLP. Glutamine at position 199 is not observed in homologous transaminases, making this non-natural substitution a novel and beneficial modification. These findings emphasize the importance of second-shell interactions in stabilizing PLP and expand our understanding of the structural diversity within PLP fold type IV transaminases. This paves the way for the engineering of more stable and versatile biocatalysts for industrial applications.

## 1. Introduction

Pyridoxal-5′-phosphate (PLP)-dependent transaminases (TAs) represent a large, diverse superfamily of enzymes that catalyze the reversible stereoselective transfer of amino groups from amino acids or amines to keto acids, ketones, and aldehydes [1,2,3]. Due to their high selectivity, TAs have attracted considerable interest in industrial asymmetric amination processes [4,5,6,7]. The D-amino acid superfamily of TAs (or TAs of PLP fold type IV according to the classification of PLP-dependent enzymes [8]) includes subfamilies of L-stereoselective branched-chain amino acid transaminases (BCATs), D-amino acid transaminases (DATAs), and (*R*)-amine–pyruvate transaminases ((*R*)-ATAs) [9,10]. The functional unit of any TA is a dimer; two active sites are formed by the residues of both subunits, with a cofactor covalently bound to the catalytic lysine residue in the *re*-position in the active site [3,10]. The domain and active site architecture of TA dimers, including the cofactor coordination, are highly conserved [9,10]. However, the active site functional residues involved in substrate coordination vary between subfamilies, resulting in different substrate specificity of TAs [9,11,12]. Earlier, the characteristic or specificity-determining motifs were revealed for the subfamilies of PLP fold type IV TAs [9,13] (Table 1).

The characteristic motifs, as originally described by Höhne et al. [13], comprise first- and second-shell residues. Here, we consider residues that interact directly with the substrate and/or PLP to be first-shell residues and residues that interact with first-shell residues and thus influence catalysis to be second-shell residues [14,15]. While the role of first-shell residues is unambiguously defined by structural analysis and mutagenesis studies [16,17,18,19,20,21,22,23,24], the effects of second-shell residues in stabilizing cofactors and modulating enzyme activity are more intriguing and challenging to explain. Noteworthily, the evolved biocatalysts (modified ATA-117) for sitagliptin synthesis contain 27 substitutions that are mostly outside the active site [4].

Several studies have emphasized the functional importance of second-shell residues in TAs. In BCATs, for example, the effective binding of the α-carboxylate group within the active site is facilitated by second-shell arginine residues, which polarize the hydroxyl group of a tyrosine residue and the main-chain NH groups that interact directly with the substrate [22]. In the ω-transaminase from *C. violaceum*, engineering efforts targeting the second coordination shell yielded a threefold increase in PLP affinity by introducing a hydrogen bond with an aspartate residue that stabilizes the N1 atom of the cofactor [25]. In porcine aspartate aminotransferase, a hydrogen bond network involving second-shell residues (namely, threonine and two histidine residues) has been shown to stabilize the interaction between the N1 atom of PLP and the conserved aspartate residue. This maintains the protonation of the N1 atom, which is essential for catalysis [26]. Structural studies of the DATA from *H. hydrossis* (Halhy) reveal the hydrogen bond network surrounding the N1 atom, including not only the conserved first-shell glutamate residue but also the second-shell arginine and asparagine residues [27]. Furthermore, mutagenesis and kinetic analysis of the variants demonstrate that the conserved second-shell arginine residue (R90 in Halhy) plays a role in maintaining the correct cofactor state [28]. Studies on (*R*)-ATAs have shown that replacing the second-shell glutamate residue with a glutamine residue alters the pH optimum of the transaminase reaction and influences the pKa of the first-shell histidine residue, which is thought to be involved in substrate deprotonation [29]. The pH dependence of the kinetic parameters of aspartate aminotransferase from *E. coli* appears to be determined by the protonation state of residues that interact with the cofactor, as well as that of some outlying residues [30].

DATAs catalyze the transfer of an amino group from a D-amino acid to a keto acid, forming a new D-amino acid and a new keto acid in the process [9]. The overall transamination reaction is a combination of two half-reactions. Firstly, the deamination of a D-amino acid results in the formation and release of a keto acid, accompanied by the amino group transfer to the cofactor. In the second half-reaction, the new keto acid enters the active site, releasing the new D-amino acid and restoring the initial PLP form of TAs [3,31]. DATAs are mainly active toward some D-amino acids and keto acids, with transamination between D-alanine and α-ketoglutarate being the most effective [32,33,34,35,36,37,38]. The active site of PLP fold type IV TAs is a combination of two pockets: the O-pocket, located near the O3′ atom of PLP, and the P-pocket, located near the phosphate group of PLP [9]. Two groups of DATAs have been discovered so far (Table 1). Group I DATAs, which include the canonical DATA from *Bacillus* sp. YM-1 (bsDATA), are characterized by a conserved Y-R-H triad in the active site (“carboxylate trap”), which ensures the binding of the α-carboxylate group in the O-pocket and therefore selectivity toward D-amino acids [39,40]. Group II (non-canonical) DATAs lack the “carboxylate trap” but instead contain a conserved R-[RK] dyad in the active site comprising a first-shell arginine residue and a second-shell arginine or lysine residue [32,33,34,35,36] (Table 1). Some group II DATAs exhibit activity toward primary (*R*)-amines [32,33]. The first-shell conserved arginine residue binds the α-carboxylate group in the O-pocket [35,41]. It also modulates PLP binding via a network of noncovalent interactions [28], and the second-shell conserved arginine residue ensures the first-shell arginine residue is positioned correctly for catalysis [28,41]. It has also been shown that the flexibility of the arginine residues favors the binding of the hydrophobic moiety of (*R*)-amines in the O-pocket of the WT DATA from *B. saxobsidence* (BlasaTA) and Halhy variant, thus providing the activity toward (*R*)-amines [28,32]. Furthermore, the second-shell arginine residues (R90 or R88 in Halhy and the DATA from *A. colombiense* (AmicoTA), respectively) contribute to the stabilization of the functional dimer [28,41].

In this study, we provide a detailed characterization of a novel non-canonical DATA from the Gram-negative bacterium *Desulfomonile tiedjei* (DestiTA) [42]. This enzyme has a unique R-D dyad in the active site, a combination of a first-shell conserved arginine residue and a second-shell aspartate residue (Table 1), while retaining activity toward D-amino acids and primary (*R*)-amines. We also observed changes in the second-shell residues of the PLP binding site. We investigated the effects of second-shell residues on PLP binding and the catalytic efficiency of DestiTA through a combination of biochemical, structural, and mutagenesis analyses. Our findings deepen our understanding of the subtle structure–function relationship in PLP-dependent TAs and suggest effective substitutions to improve their stability.

## 2. Results

### 2.1. Enzyme Identification, Expression, and Purification

Searching for non-canonical DATAs revealed a TA from the Gram-negative bacterium *D. tiedjei*—DestiTA Uniprot ID I4C827_DESTA (Table 1). DestiTA shared 25.4% sequence identity with the BCAT from *E. coli*; 24.0% with the (*R*)-ATA from *A. fumigatus*; 27.4% with the bsDATA *from Bacillus* sp., and 23–32% with group II DATAs. DestiTA was cloned and expressed in a soluble form in *E. coli* and purified to homogeneity (Figure S1). The molecular weight of purified DestiTA was approximately 68 kDa, as determined by gel filtration, indicating DestiTA as a homodimer (Figure S2).

### 2.2. Substrate Specificity, Enantioselectivity, and Stability

In the overall transamination reactions, DestiTA was active toward some D-amino acids and α-keto acids (Table S1). No activity toward L-amino acids was detected. DestiTA was also active toward (*R*)-(+)-1-phenylethylamine ((*R*)-PEA) but not toward (*S*)-PEA. The specificity constant for (*R*)-PEA, however, was 150 times lower than that for D-alanine (Table 2). The activity toward ketones was not observed (characteristic feature of (*R*)-ATA [11,13]). The enantioselectivity of DestiTA was evaluated in the amination of 3-methyl-2-oxobutyrate with D-glutamate as an amino donor. The product yield of D-valine after 24 h of the reaction at 40 °C achieved 99%. The enantiomeric excess of D-valine exceeded 99% (Table S2). Thus, DestiTA was ascribed to group II DATAs based on the sequence analysis (Table 1) and substrate scope.

**Figure 1:**
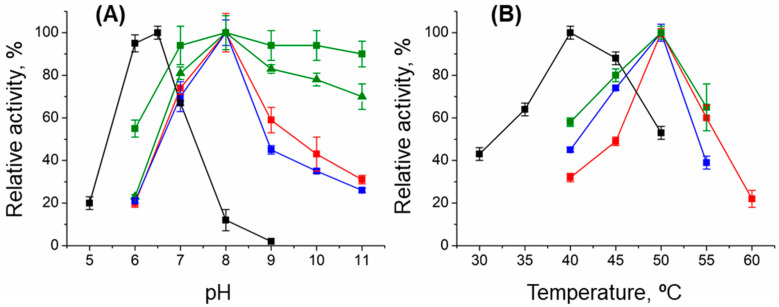
The pH (**A**) and T dependences (**B**) of the relative activity of the WT (red), T43E (black), T199N (blue), and T199Q (green) variants in the overall transamination reaction between 25 mM D-alanine and 10 mM α-ketoglutarate. In (**A**), 100% corresponds to 4.5 ± 0.2 for WT; 0.10 ± 0.01 for T43E; 4.0 ± 0.2 for T199N; and 2.7 ± 0.2 (25 mM D-alanine, ■) and 4.5 ± 0.4 U/mg (100 mM D-alanine, ▲) for T199Q at 40 °C in mixed buffer containing 25 mM Na-acetate, 25 mM Na-phosphate, and 25 mM Na-pyrophosphate. In (**B**), 100% corresponds to 11.5 ± 0.2 for WT; 0.16 ± 0.01 for T43E; 8.3 ± 0.3 for T199Q; and 4.0 ± 0.1 U/mg for T199N in 50 mM K-phosphate buffer, pH 8.0 (WT, T199N, T199Q) or pH 6.0 (T43E). Bars mean standard deviation.

pH dependences of DestiTA-specific activities are typical of TAs: the pH range of 7.5–8.0 is optimal for the reaction *D-alanine + α-ketoglutarate* (Figure 1), and the pH range 8.0–9.0 is the best for the reaction *(R)-PEA + α-ketoglutarate* (Figure S3). Further kinetic analysis with D-alanine and (*R*)-PEA was performed in 50 mM K-phosphate buffer, pH 8.0, at 50 °C, and in 50 mM Na-pyrophosphate buffer, pH 9.0, at 50 °C, respectively. The stability of DestiTA was examined by determining its half-transition temperature (T_0_._5_), representing the midpoint between its native and denatured states during thermal unfolding of the apoenzyme and holoenzyme (Table 2).

**Figure 2:**
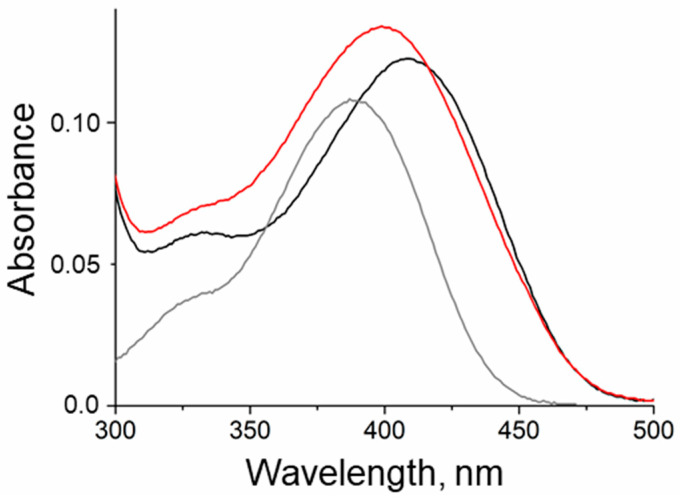
The absorption spectra of 15 µM holoenzyme of DestiTA before (black) and after five min of incubation (red) in 50 mM K-phosphate buffer, pH 8.0, at 50 °C. The grey line represents the spectrum of 15 µM free PLP.

The stability of the holoenzyme was evaluated by analyzing spectra and determining the equilibrium dissociation constant (K_d(PLP)_) of the holoenzyme as an indicator of DestiTA’s affinity to the cofactor. DestiTA’s absorption spectrum exhibited two distinctive peaks: a dominant peak at 280 nm and a prominent peak at 410 nm, accompanied by a shoulder at 330 nm (Figure 2). The 410 nm band corresponded to the ketoenamine tautomer of the protonated internal aldimine, while the shoulder at 330 nm corresponded to the enolimine tautomer [43]. Monitoring changes to the absorption spectrum of the DestiTA holoenzyme upon incubation in a 50 mM K-phosphate buffer, pH 8.0, at 50 °C for five minutes revealed a shift in the 410 nm peak to 399 nm, indicating PLP leakage and suggesting instability of the holoenzyme under these conditions. The affinity of DestiTA to PLP was further quantified by a thermal shift assay, which yielded a K_d(PLP)_ value of 11 ± 1 µM. This is approximately fivefold higher than the reported value for the homologous Halhy [28], suggesting weaker PLP binding. A structural analysis of the PLP binding site and the active site architecture of the DestiTA dimer was carried out to evaluate the holoenzyme’s stability.

### 2.3. Overall Structure of DestiTA. PLP Coordination in the Active Site

Unfortunately, all attempts to crystallize WT DestiTA and its variants were unsuccessful. NMR spectroscopy, a powerful technique for determining protein structures in solution, will be considered in future work. A DestiTA model was created for structural analysis.

**Figure 3:**
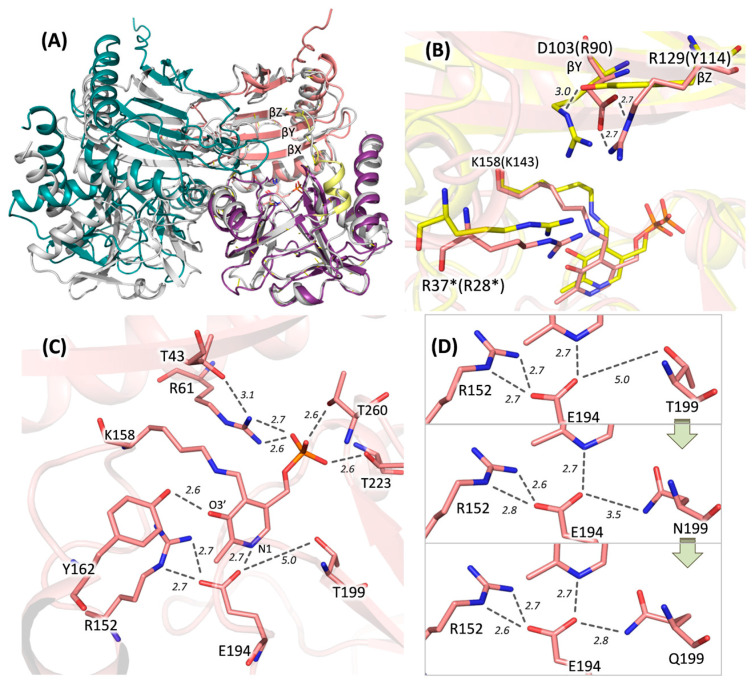
Model of the DestiTA functional dimer. (**A**) The superposition of the DestiTA model (the left subunit is colored in green; in the right subunit, the small domain is colored in pink, the large domain in purple, and the interdomain loop in yellow) and the crystal structure of Halhy (grey, PDB ID: 7P7X). (**B**) The superposition of the active sites of the DestiTA model (pink) and Halhy (yellow, 7P7X). (**C**) Binding PLP in the active site of DestiTA. (**D**) Changes in the noncovalent bond network around the N1 atom of PLP upon the T199N and T199Q substitutions. (*) indicates residues from the adjacent subunit. The distances are given in Å.

Based on gel filtration data, DestiTA was modeled as a homodimer. The DestiTA subunit model included all structural elements characteristic of PLP fold type IV TAs observed in crystal structures (Figure 3A). A structural comparison of the DestiTA model and the AmicoTA–D-glutamate complex (PDB ID: 8AYK) revealed key residues involved in substrate binding. The α-carboxylate group of the substrate was likely coordinated by residues R37* and T197 in the O-pocket. Meanwhile, the γ-carboxylate group of either D-glutamate or α-ketoglutarate appeared to interact with residue R262 in the P-pocket (see Figure S4).

A distinctive feature of DestiTA, compared to other group II DATAs, was the presence of a negatively charged D103 residue in the O-pocket of the active site (see Table 1 and Figure 3B). Detailed inspection revealed a single plausible interaction involving D103: the formation of a salt bridge with R129, which is located on the βZ-strand (Figure 3B). This interaction positioned D103 away from the active site, with its side chain located ~7 Å from the first-shell residue R37*, which was too distant for interaction—a prerequisite for a second-shell residue. In the homologous Halhy and AmicoTA, the distances between the conserved second-shell and first-shell arginine residues (the latter of which is crucial for coordinating the substrate’s α-carboxylic group) were 3.6 and 3.7 Å, respectively (Figure 3B). Consequently, R37* appeared to be less constrained by neighboring residues than in Halhy and AmicoTA.

**Figure 4:**
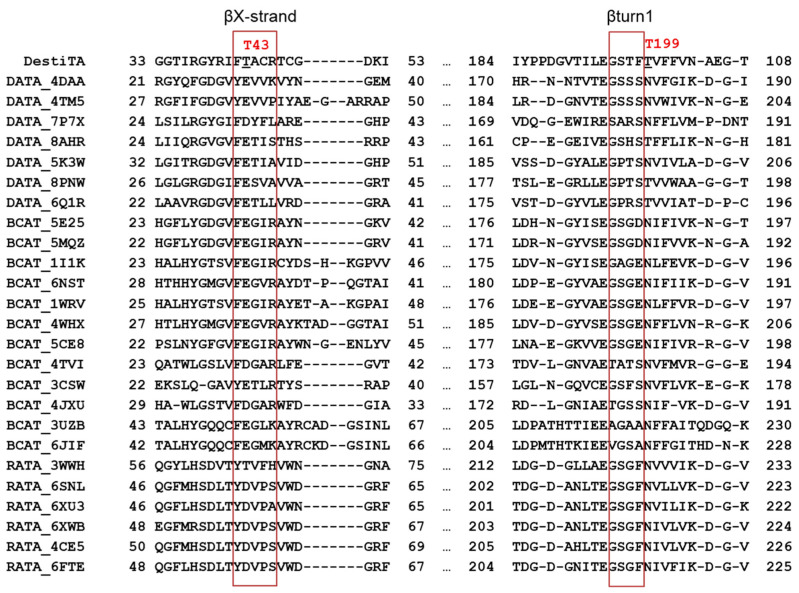
Structure-based sequence alignment of DestiTA and TAs of PLP fold type IV with known structures, referred to by PDB codes.

We analyzed the PLP-binding site in DestiTA. The PLP molecule was covalently bound to the K158 residue and coordinated by residues that are highly conserved and typical of PLP fold type IV TAs. E194 and Y162 interacted with the N1 atom and the O3′ atom of PLP, respectively. Meanwhile, R61, T223, and T260 coordinated the phosphate group (see Figure 3C). However, DestiTA differed from the known PLP fold type IV TAs in the second coordination shell of the PLP binding site (see Figure 4).

The first notable difference was the replacement of a conserved acidic residue (glutamate or aspartate) on the βX-strand with a threonine residue (T43). In the listed TA structures (except the (*R*)-ATA from *Arthrobacter* sp. KNK168, PDB ID: 3WWH), a hydrogen-bonded salt bridge was observed between the side chains of the glutamate/aspartate residue and the arginine residue that interacted with the phosphate group of PLP (Figure S5). The glutamate residue (at position T43 in DestiTA) appeared to be important for PLP binding, as was demonstrated earlier for bsDATA [44].

Another hot spot was observed near the glutamate residue coordinating the N1 atom of PLP. In Halhy, a hydrogen-bonding network involving E176, R137, and N181 contributed to the stabilization of the protonated N1 atom (Figure S5) [27]. In contrast, in DestiTA, while the conserved R152 was retained, the position homologous to N181 was occupied by a threonine residue (T199) (Figure 3C). Generally, only threonine or asparagine residues were found in this position in homologous TAs (Figure 4 and Table S3). In structures containing a threonine residue, the distance between its side chain and the γ-carboxylic group of the glutamate residue coordinating the N1 atom of PLP (E/OE1–T/OG1 bond) was greater than 4.5 Å. This is too large for a hydrogen bond to form (Table S3). In contrast, in structures with an asparagine residue, the corresponding distance (E/OE1–N/ND2 bond) decreased to 3.1–3.4 Å, enabling the formation of a weak hydrogen bond. Based on this analysis, we selected the T199N and T199Q substitutions for experimental study. T199Q was a non-natural substitution: the longer side chain of the glutamine residue compared to the asparagine residue may favor a stronger hydrogen bond with the N1-coordinating glutamate residue, thus potentially enhancing the cofactor binding. Further structural modeling of the T199N and T199Q variants revealed a progressive shortening of the distance between the side chains of E194 and residue 199 from 5.0 Å in the WT to 2.8 Å in the T199Q variant, suggesting the strengthening of the stabilizing hydrogen bond network (Figure 3D).

A previous study of Halhy demonstrated the input of the second-shell arginine (R90, see Table 1) in stabilizing the PLP molecule [28]. However, substituting D103 for arginine would result in electrostatic repulsion with R129, disrupting the active site. Therefore, we ruled out the D103R substitution.

Overall, we chose the T43E, T199N, and T199Q substitutions to improve PLP binding. These variants were expressed in a soluble form in *E. coli* and purified to homogeneity (Figure S1). Gel filtration confirmed the homodimer organization of the variants (Figure S2). Prior to examining the effects of the substitutions on PLP binding, we performed a general characterization of the variants.

### 2.4. Characterization of the DestiTA Variants: Thermal Stability and Catalytic Function

The T43E substitution had a dramatic impact on catalytic activity. The variant showed no activity in the reaction *D-alanine + α-ketoglutarate* at pH 8.0 and retained only 2% of WT activity at pH 6.5 (Figure 1A, Table 2); the activity toward (*R*)-PEA was lost. Additionally, the temperature optimum shifted to 40 °C (Figure 1B). The T199N substitution caused no changes in the catalytic properties of DestiTA in the reaction between D-alanine and α-ketoglutarate (Table 2, Figure 1). Meanwhile, the T199Q substitution caused a ~100-fold decrease in the specificity constant (*k_cat_*/*K_m_*) for D-alanine but did not affect the catalytic constant (*k_cat_*), indicating a decrease in substrate binding affinity of DestiTA (Table 2). The catalytic efficiency toward α-ketoglutarate decreased only slightly, possibly due to its multipoint binding in the active site. The T199N and T199Q variants were both inactive toward (*R*)-PEA.

Interestingly, the T199Q substitution significantly improved the pH stability of DestiTA. The T199Q variant’s pH activity profile was broader than those of WT and T199N for the *D-alanine + α-ketoglutarate* reaction (Figure 1A). The T199Q variant retained 70–90% of its activity at alkaline pH values for both sub-saturating (25 mM) and near-saturating (100 mM) concentrations of D-alanine (Figure 1A).

We assessed the thermal stability of the DestiTA variants using the half-transition temperature (T_0.5_) (Table 2). Similar to WT DestiTA, the thermal stability of the T199N and T199Q variants depended on the presence of PLP in the assay buffer. Adding a saturating amount of PLP to the apoenzyme increased T_0.5_ by over 8 °C. The T199Q substitution improved the stability of the holoenzyme; however, the stability of the apoenzyme decreased. The T43E substitution increased the T_0.5_ of the apoenzyme by 5 °C, likely due to the formation of a stabilizing salt bridge between the side chains of E43 and R61 (Figure S6). Notably, the thermal stability of the T43E variant remained unaffected by the addition of PLP.

In summary, introducing a negatively charged residue at position 43 led to the loss of the catalytic function of DestiTA, while substitution at the 199 position to glutamine, but not to asparagine, affected substrate binding affinity and pH stability.

### 2.5. Characterization of the DestiTA Variants: Cofactor Binding and Internal Aldimine Formation

To examine the effects of the substitutions of the PLP binding, we investigated the cofactor binding by the thermal shift assay and the internal aldimine state by CD and UV-Vis spectroscopy.

**Figure 5:**
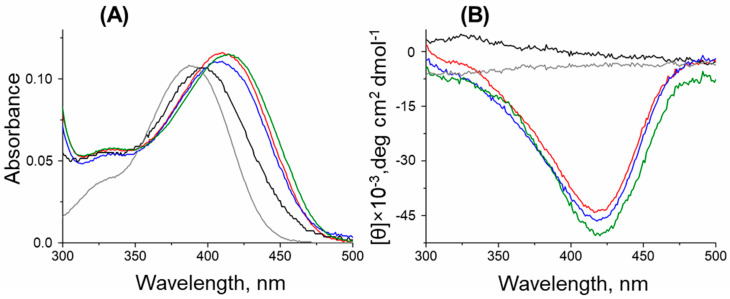
Spectral analysis of the internal aldimine of the WT Desti (red), T43E (black), T199N (blue), and T199Q (green) variants in 50 mM K-phosphate buffer, pH 8.0, at 25 °C. (**A**) Absorbance spectra of 15 µM holoenzymes of the variants. (**B**) CD spectra of 15 µM apoenzymes of the DestiTA variants in the presence of 30 µM PLP (1 mM in the case of the T43E variant). Gray lines represent spectra of free PLP (15 µM in **A**; 30 µM in **B**).

The holoenzymes (PLP forms) of the T199N and T199Q variants exhibited spectral features similar to the WT variant (Figure 5A). Since the pH dependence of the DestiTA activity changed with the T199Q substitution, we checked if this substitution affected the protonation state of the Schiff base under alkaline conditions. The pH titration experiment revealed that both the WT and T199Q variants had a protonated internal aldimine in solution at pH 8–10. This was evident by the absence of an absorption maximum at 360–380 nm, which is typically associated with the deprotonated aldimine form (Figure S7) [23,44]. The absorbance spectrum of the T43E variant, incubated with an excess of PLP, indicated the unbounded state of PLP (Figure 5A).

The thermal shift assays revealed K_d(PLP)_ values of 33 ± 2 µM for the T199N variant and 2.2 ± 0.4 µM for the T199Q variant at pH 8.0 in 50 mM K-phosphate buffer, indicating a fivefold increase in PLP affinity for T199Q relative to WT. This enhanced affinity was maintained at higher pH values: at pH 10.0, the K_d(PLP)_ values were 21 ± 4 µM for WT and 1.4 ± 0.3 µM for T199Q; at pH 11.0, the values were 20 ± 4 µM for WT and 1.2 ± 0.2 µM for T199Q. The thermal shift upon adding PLP to the T43E solution was not observed.

We utilized circular dichroism (CD) spectroscopy to detect the internal aldimines in an excess of free PLP, which lacks the CD signal [45,46]. The WT, T199N, and T199Q variants exhibited a similar strong negative dichroic signal at 420 nm, which is indicative of internal aldimine formation (Figure 5B). However, no CD signal was detected for the T43E variant, even with 1 mM excess PLP, suggesting an impaired ability to form a functional internal aldimine (Figure 5B). Notably, adding increasing concentrations of PLP (up to 600 µM) to the reaction mixture did not enhance the catalytic activity of the T43E variant (Table S4), thus showing that its reduced activity was not due to cofactor insufficiency. Together with the absence of the thermal shift, these observations show the inability of the T43E variant to form active holoenzymes (PLP form).

## 3. Discussion

Group II DATAs are primarily distinguished by the conserved dyad within the characteristic motifs of the active site: a critical arginine residue (first-shell) that binds the substrate’s α-carboxylate group, paired with a second-shell positive residue (R or K) (Table 1). Interestingly, DestiTA retains only the conserved arginine residue (R37) in the first shell, while the second position is occupied by a negatively charged aspartate residue (D103). This combination is typical of a subfamily of (*R*)-ATAs [11]; however, DestiTA does not catalyze the amination of ketones or the deamination of (*R*)-primary amines but rather the deamination of (*R*)-PEA. The latter is an unspecific substrate for some BCATs [47,48] and some group II DATAs [28,32]. Thus, DestiTA exhibits the structure–function relationship characteristic of group II DATAs, showing activity toward D-amino acids, α-keto acids, and (*R*)-PEA. In terms of catalytic efficiency, DestiTA is second only to Halhy. This finding indicates the structural variability within group II DATAs, and we hypothesize that substituting the second-shell arginine residue with an aspartate residue (D103) enhances the conformational flexibility of the first-shell arginine residue (R37), thus facilitating the binding of the hydrophobic moiety of (*R*)-PEA within the O-pocket and supporting the well-known promiscuity of TAs [49]. This is consistent with previous findings: in BlasaTA, which is active toward some primary aromatic (*R*)-amines, the flexibility of the conserved first-shell arginine residue appears to be intrinsic; in Halhy, substitution of the second-shell arginine residue induces side-chain flexibility in the first-shell arginine residue, thus favoring (*R*)-PEA activity [28,32]. At the same time, D103 may also decrease holoenzyme stability, as evidenced by the higher value of the PLP dissociation constant value of WT DestiTA compared to Halhy [28].

PLP binding is conserved in TAs of PLP fold type IV. The cofactor is covalently bound within the active site via a Schiff base linkage to a catalytic lysine residue, forming an internal aldimine. Four functional groups of PLP—the phenolic oxygen (O3′ atom), phosphate group, pyridine nitrogen (N1 atom), and Schiff base—are essential for the proper binding of PLP in the active site. Their protonation states are controlled by the local environment, especially through specific hydrogen bonds and electrostatic interactions provided by surrounding amino acid residues [31,50]. Thus, the protonation of the N1 atom, which is critical in enhancing the electron sink capability of PLP, thereby facilitating transamination, is stabilized by a hydrogen bond with a conserved aspartate or glutamate residue and second-shell residues [23,24]. The phenolic oxygen (O3′ atom) typically forms one or two hydrogen bonds with neighboring proton-donating side chains, contributing to proper positioning of the cofactor and its ionization state [18,21,22]. Disruption of these interactions has been shown to impair catalytic function, likely due to destabilizing PLP’s electronic properties and altering the ionization state of the Schiff base [17,18,19,20]. The phosphate group in the active site of TAs is coordinated by an arginine residue and several polar residues; the lack of one of these bonds leads to the impairment of PLP binding [16]. Although the primary coordination of PLP—via interactions with K158 (Schiff base), E194 (N1), Y162 (O3’), and R61/T223/T260 (phosphate group)—is conserved in DestiTA, structural comparisons with homologous enzymes revealed some differences in the second-shell environment that seem to contribute to the observed weaker PLP binding.

Two positions—T43 and T199—were selected for mutation analysis to improve PLP binding. Position 43 is located near the phosphate group of PLP. Despite the presence of a glutamate residue at this position in homologous enzymes, the T43E substitution in DestiTA drastically reduced the enzyme activity and impaired internal aldimine formation. Introducing a negatively charged glutamate in this area, particularly near the catalytic lysine (see Figure 3C), appears to disturb the finely tuned electrostatic environment required for proper PLP anchoring in DestiTA. Furthermore, the formation of a salt bridge between the catalytic lysine cannot be ruled out. It leads to the instability of the holoenzyme and the removal of the catalytic lysine from the active site. This unfavorable salt bridge may be partly neutralized at an acidic pH due to the protonation of the E43 side chain, which shifts the pH optimum to the acidic region. The instability of the T43E holoform may also lead to a decrease in the optimum temperature of the transamination reaction. These findings contrast with mutation experiments involving bsDATA, whereas the opposite substitutions at the homologous position (E32Q and E32A) led to a loss of activity [44]. Together, these results highlight the symbiotic (compensating) effects of hydrogen bonds coordinating PLP in TAs, particularly when second-shell residues contribute to shaping the electronic and structural environment of the cofactor.

Interesting findings emerged from the analysis of position 199, located near the N1 atom of PLP. While both T199N and T199Q substitutions preserve the structural features necessary for catalytic function, only the T199Q variant enhances cofactor affinity. Threonine and asparagine residues are equivalent in this position. Importantly, a glutamine residue at position 199 is not observed in any known homologous PLP fold type IV TAs. Introducing a glutamine at position 199 represents a non-natural but beneficial substitution. Moreover, the T199Q substitution favors DestiTA activity in the alkaline region. Generally, shifting the pH optima of TAs remains a significant challenge in enzyme engineering, with only a few successful examples reported thus far [29,41,51]. Spectral analysis of the T199Q variant revealed the protonated state of the internal aldimine at alkaline pH values. Furthermore, the T199Q variant exhibits tight PLP binding across the pH range of 8 to 11. It appears that the T199Q substitution increases the stability of the protonated state of the N1 atom of PLP, thus favoring the formation of the transamination reaction intermediates, particularly the quinonoid intermediate, at neutral and alkaline pH values. The stability of the quinonoid intermediate favors the enzyme activity in alkaline media, where the concentration of protons decreases. This substitution stabilizes the holoenzyme, resulting in a higher T_0.5_.

Engineering strategies for PLP-dependent enzymes generally focus on stabilizing the active enzyme in water–organic solvent media with high substrate concentrations at temperatures between 40 and 60 °C [9,52]. For instance, sitagliptin production was most efficient at 50% dimethyl sulfoxide, pH 8.5, 1 M isopropanol, and 0.5–1 g/L PLP at 40 °C [4]. Improving PLP binding in the holoenzyme is not of prime importance when developing PLP-dependent biocatalysts. Typically, PLP leakage from the holoenzyme is solved by adding PLP in excess to the reaction medium. More important is the thermal stability of the biocatalyst, including its operational stability. It is also clear that stabilizing PLP in the active holoenzyme improves its operational stability in limited PLP conditions and its thermal stability as well [25,53]. The thermal and operational stabilities of DestiTA in limited PLP conditions were affected by stabilizing the protonation state of the N1 atom of PLP. This was achieved by substituting the threonine residue for the glutamine residue, which is unusual for this position in TAs. This was demonstrated for TAs for the first time.

## 4. Materials and Methods

### 4.1. Preparation and Purification of the Recombinant DestiTA and Its Variants

The gene *Desti_3054* encoding a fold type IV transaminase was found in the complete genome sequence of *Desulfomonile tiedjei* DSM 6799 [42]. The nucleotide sequence of *Desti_3054* was codon-optimized for expression in *E. coli* using server *Optimizer* (http://genomes.urv.es/OPTIMIZER/ (accessed on 2 August 2024)) and synthesized by ATG Service Gene (St. Petersburg, Russia) with overhangs complementing the *Nde*I and *Hin*dIII restriction sites attached to the 5′- and 3′-ends, respectively. The synthetic gene was cloned into the pET-21d vector (Novagen, Darmstadt, Germany), modified as described in [28]. Recombinant DestiTA (302 a.a., 34.2 kDa) fused at the N-terminus to a (His)6-TEV-tag was expressed in *E. coli* Rosetta(DE3) pLysS cells (Novagen). The transformed cells were grown in LB medium, supplemented with 100 µg/mL ampicillin and 20 µg/mL chloramphenicol (Panreac-AppliChem, Darmstadt, Germany) and 0.04 mM PLP at 37 °C until the OD600 value reached 0.8, and the expression was induced with 0.2 mM IPTG. After incubation for 18 h at 24 °C, the cells were harvested by centrifugation, resuspended in 50 mM Na-phosphate buffer, pH 8.0, supplemented with 500 mM NaCl, 20 mM imidazole, 2 mM β-mercaptoethanol, 0.1% (*v*/*v*) Triton X-100, 10% (*v*/*v*) glycerol, 2 M urea, 0.2 mg/mL lysozyme, 100 µM PLP, and 1 mM PMSF, and disrupted by sonication. The crude cell extract was centrifuged for 45 min at 18,500× *g*. The supernatant was loaded into a HisTrap HP column (Cytiva, Marlborough, MA, USA), equilibrated in a binding buffer (50 mM Na-phosphate, pH 8.0, supplemented with 500 mM NaCl, 20 mM imidazole, 10% (*v*/*v*) glycerol, and 0.1% (*v*/*v*) Triton X-100). The (His)6-tagged recombinant DestiTA was eluted with a linear gradient from 20 to 500 mM imidazole in the same buffer without Triton X-100. The active fraction was incubated for 1 h with a twofold molar excess of PLP at +4 °C and then concentrated using a 30 kDa cut-off centrifugal filter device (Millipore, Burlington, MA, USA) and transferred into 50 mM Tris-HCl buffer, pH 8.0, supplemented with 50 mM NaCl, 1 mM EDTA, 5 mM β-mercaptoethanol, 10% (*v*/*v*) glycerol, and TEV protease (1 mg per 10 mg of the protein). The solution was incubated for 2 h at room temperature, dialyzed against the binding buffer, and loaded into a HisTrap HP column. The flow-through was concentrated and applied to a Superdex 200 10/300 GL column (Cytiva) equilibrated in 20 mM HEPES buffer, pH 8.0, supplemented with 100 mM NaCl and 100 µM PLP. Active fractions of DestiTA were collected and stored at +4 °C. The protein purity was detected by SDS-PAGE (12%). The protein concentration was determined spectrophotometrically [54].

The T43E, T199N, and T199Q variants of DestiTA were created by easy single-primer site-directed mutagenesis as described in [55]. The list of the mutagenesis and check primers is given in Table S5. All variants were expressed in a soluble form in *E. coli* and purified to homogeneity as described for the WT DestiTA. The amino acid sequences were verified by MALDI-TOF MS analysis (UltraFlextreme Bruker Daltonik, Bremen, Germany).

The PLP forms of the DestiTA variants were obtained by incubating the enzymes with a tenfold molar excess of PLP and 5 mM α-ketoglutarate for 2 h at 25 °C, followed by transfer into the assay buffer, pH 8.0, using a HiTrap desalting column (GE Healthcare, Chicago, IL, USA).

The DestiTA variant apoenzymes were obtained by incubating the PLP forms with 2 mM phenylhydrazine for 20 min at 25 °C, followed by transfer into the assay buffer, pH 8.0, using a HiTrap desalting column.

### 4.2. Enzyme Activity Assay

The activity of the DestiTA variants in the transamination reaction with D-alanine or D-glutamate as the amino donor was observed spectrophotometrically using the second enzymatic reaction with lactate dehydrogenase from rabbit muscle (LDH) (Roche Diagnostic GmbH, Mannheim, Germany) or (*R*)-2-hydroxyglutarate dehydrogenase (HGDH) from *Acidaminococcus fermenats* [36] using NADH as the coenzyme. The reaction progress was monitored by the absorbance decay at 340 nm (ε_NADH_ = 6.22 mM^−1^cm^−1^) in microtiter plates (UV-Star, Greiner, Germany) using a SPECTROstar Omega plate reader (software version 5.50 R4, BMG Labtech, GmbH, Ortenberg, Germany). The activity of the DestiTA variants in the transamination reaction with (*R*)-(+)-1-phenylethylamine ((*R*)-PEA) as the amino donor was determined by monitoring acetophenone (AcPh) production at 245 nm (ε_AcPh_ = 11.6 mM^−1^ cm^−1^), using an Evolution 300 UV-Vis spectrophotometer (Thermo Scientific, Waltham, MA, USA). The activity of the variants was calculated from the initial linear region of the reaction’s progress curve. One unit (U) was defined as the amount of the enzyme that catalyzed the conversion of 1 μmol of substrate into a product per minute.

The reaction with D-alanine was conducted in 50 mM K-phosphate buffer, pH 8.0 or 6.5, supplemented with 0.5–200 mM D-alanine, 0.1–50 mM α-ketoglutarate, 30 μM PLP, 2–200 μg/mL (0.05–5 μM) of the purified variant, 330 μM NADH, and 2 U/mL LDH at 40–50 °C. The reaction with (*R*)-PEA was observed in 50 mM pyrophosphate buffer, pH 9.0, supplemented with 1–100 mM (*R*)-PEA, 0.1–10 mM α-ketoglutarate, 30 μM PLP, and 0.036–0.1 mg/mL (1–2.5 μM) of the purified variant at 50 °C.

The kinetic parameters of the overall reactions were determined using the Michaelis–Menten model. The substrate saturation curves were generated at constant co-substrate concentrations to calculate the kinetic parameters (Table S6). The kinetic parameters were calculated by fitting the initial velocity data to Equation (1):

(1) $$V=\frac{V_{max}\times A\times B}{K_{m}^{A}\times B+K_{m}^{B}\times A+A\times B}$$

where *V* is the initial velocity, *V_max_* is the maximal velocity, A and B are the substrate concentrations, and $K_{m}^{A}$ and $K_{m}^{B}$ are the *K_m_* of substrates A and B, respectively. All measurements were performed at least in triplicate. The data were analyzed using Origin 8.0 software (Origin Lab, Northampton, MA, USA, assessed on 14 May 2025).

### 4.3. Effect of pH and Temperature on the Transamination Reaction

pH and temperature effects were examined using overall transamination reactions between 10 mM α-ketoglutarate and 25 (or 100 mM) D-alanine or 2 mM α-ketoglutarate and 5 mM (*R*)-PEA. The optimal pH was determined at 40 °C in a mixed buffer of 25 mM Na-acetate, 25 mM Na-pyrophosphate, and 25 mM Na-phosphate, pH 6.0–11.0. The effect of temperature on the reaction rate was analyzed in 50 mM K-phosphate buffer, pH 8.0 or 6.5, or 50 mM Na-pyrophosphate, pH 9.0.

### 4.4. Spectral Analysis

Absorption spectra were collected in a 1 cm optical path length cuvette using an Evolution 300 UV–Vis spectrophotometer. CD spectra were collected in a 1 cm optical path length cuvette using a Chirascan instrument (Applied Photophysics, Surrey, UK).

### 4.5. Analysis of the Enantiomeric Excess in the Transamination Reaction

The enantioselectivity of DestiTA was evaluated in the amination reaction *3-methyl-2-oxobutyrate + D-glutamate*. To shift the equilibrium toward the products, a one-pot three-enzyme system was employed. The coproduct, α-ketoglutarate, was removed from the reaction mixture using HGDH, while NADH was recovered by glucose dehydrogenase (GDH) (Sigma, St. Louis, MO, USA) converting D-glucose. The reaction mixture contained 100 mM K-phosphate buffer, pH 7.5, 100 µM PLP, 50 mM D-glutamate, 50 mM 3-methyl-2-oxobutyrate, 0.1 mg/mL DestiTA, 50 μM NAD+, 150 mM D-glucose, 180 U/mL HGDH, and 30 U/mL GDH. After 24 h at 40 °C, the reaction was terminated by removing the enzyme using a 30 kDa cut-off centrifugal filter device (Millipore). The deproteinized aliquot was analyzed by HPLC (ÄKTA Purifier, Cytiva, Marlborough, MA, USA) using a reverse-phase C18 column (Zorbax Eclipse XDB-C18, 5 µm, 4.6 mm × 150 mm (Agilent, Santa Clara, CA, USA)). The chiral analysis of the D-valine was performed by HPLC using a reverse-phase C18 column with a UV detector set at 340 nm after derivatization with Marfey’s reagent (Sigma). The HPLC and derivatization conditions are shown in the Supplementary Materials (Table S2).

### 4.6. ThermoFluor Assay

The thermal stability of the WT DestiTA and the variants was evaluated by the ThermoFluor assay. The assay was conducted in 50 mM K-phosphate buffer, pH 8.0, in the presence of 25× ProteOrange Protein Gel Stain (Lumiprobe, Westminster, MD, USA). The final concentration of the enzyme was 0.07 mg/mL (2 µM). Measurements were performed using a CFX96 RT-PCR system (Bio-Rad, Hercules, CA, USA) at every 0.2 °C increment after equilibrating for 10 sec from 25 to 90 °C, with excitation and emission filters 515–530 and 560–580 nm, respectively. All thermal unfolding curves were measured in triplicate. Raw data were collected with CFX Manage software (version 2.3, Bio-Rad, Hercules, CA, USA. Assessed on 16 June 2025) and processed with Origin 8.0 software (assessed on 14 May 2025). The midpoint temperature of thermal denaturation (T_0.5_) was determined from the peak of the first derivative of the thermal unfolding curves.

### 4.7. Determination of the Equilibrium Dissociation Constant of the Holoenzyme

The dissociation constant (K_d(PLP)_) of the holoenzyme was assessed by the thermal shift assay (Figure S8). For that, the apoenzyme (2 µM) was incubated at 25 °C in the presence of different PLP concentrations of 0.5–500 µM for 1 h in either 50 mM K-phosphate buffer (pH 8.0) or 50 mM Na-pyrophosphate buffer (pH 10.0 or 11.0). The samples were then analyzed by the ThermoFluor assay using the same buffer conditions. The dissociation constant was determined by fitting the data to Equation (2):

(2) $${\Delta T}_{0.5}={\Delta T}_{0.5max}\times \frac{\left[E\right]+\left[PLP\right]+K_{d\left(PLP\right)}-\sqrt{{\left(\left[E\right]+\left[PLP\right]+K_{d\left(PLP\right)}\right)}^{2}-4[E][PLP]}}{2[E]}$$

where [E] and [PLP] represent the total concentrations of the apo form and PLP, respectively, ${\Delta T}_{0.5}$ is an observed shift in the midpoint temperature of thermal denaturation at the PLP concentration [PLP], and ${\Delta T}_{0.5max}$ is the maximum shift when all enzyme molecules are complexed with PLP. All measurements were performed at least in triplicate, and the data were analyzed using Origin 8.0 software (assessed on 14 May 2025).

### 4.8. Molecular Modeling and Structure Analysis

The in silico models of the WT, T43E, T199N, and T199Q DestiTA variants were obtained using the AlphaFold3 structure prediction server [56], followed by the energy minimization of the TA-cofactor complexes. The AlphaFold3-predicted structures consisted of two subunits without a cofactor. Coordinates for PLP molecules were added to these models based on an analysis of known crystal structures of the PLP-form DATA. The holoenzyme’s models were relaxed with NAMD Molecular Dynamics Software, Version 2.14 (Theoretical and Computational Biophysics Group, Beckman Institute, University of Illinois, Urbana, IL, USA, accessed on 22 June 2025) [57]. A protein macromolecule description was created using the topology and parameters for the CHARMM36 force field [58]. The protonation states of the amino acid residues were as follows: positively charged R and K; negatively charged E and D; and neutral H. The topology of the PLP molecule was obtained using the CHARMM General Force Field (CGenFF), Version 3.0, https://www.charmm-gui.org/, accessed on 12 January 2025 (Im Lab, Lehigh University, Bethlehem, PA, USA,) (Table S7) [59]. The parameters for the PLP were partially obtained on the aforementioned web server, and parameters were also adopted from source [60] and empirically selected. A special patch in the topology files was used to describe the Schiff base between the PLP and K158. The phosphate group of the PLP was doubly negatively charged, the O3′ atom was deprotonated, and the Schiff base was protonated. The system was then solvated and ionized. The geometry configuration of the model system was optimized for 20,000 steps.

The visual inspection of the modeled structures and figure preparation were carried out by the PyMOL Molecular Graphics System, Version 2.4. (Schrödinger, New York, NY, USA, assessed on 14 May 2025). Multiple structural alignment was performed using MUSTANG: A Multiple Structural alignment algorithm, Version 3.2.4 (Arun Konagurthu, Monash University, Australia, accessed on 16 March 2025) [61].

## 5. Conclusions

The PLP-dependent fold type IV transaminase from *Desulfomonile tiedjei* (DestiTA) was functionally characterized, including a detailed analysis of the contribution of second-shell residues to PLP binding and holoenzyme stability. Although DestiTA belongs to group II DATAs, in the catalytically important dyad, it retains only the first-shell conserved arginine residue (R37), while the second position is occupied by a negatively charged aspartate residue (D103). This combination is typical of a subfamily of (*R*)-ATAs; however, DestiTA does not catalyze the amination of ketones. DestiTA exhibits activity toward some D-amino acids and (*R*)-phenylethylamine. Site-directed mutagenesis of residues in the second shell near the PLP-binding site was performed based on structural modeling and sequence analysis of homologous transaminases. The T43E substitution in DestiTA drastically reduced the enzyme activity and impaired internal aldimine formation. While the T199N and T199Q substitutions both preserve the structural features necessary for catalytic function, only the T199Q variant enhances cofactor affinity. The T199Q variant demonstrated a fivefold increase in PLP affinity and improved activity under alkaline conditions. This was attributed to a newly formed hydrogen bond that stabilized the N1-binding region of PLP. Glutamine at position 199 was not observed in homologous transaminases, making this non-natural substitution a novel and beneficial modification. Our findings underscore the contribution of second-shell residues in stabilizing PLP and promoting the formation of the catalytically active internal aldimine in DestiTA. These results could be useful for the engineering of PLP fold type IV TAs with improved cofactor retention and functionality under industrial conditions.

## Figures and Tables

**Table 1:** The characteristic amino acid motifs in the active sites of members of subfamilies of PLP fold type IV TAs. DATA from *Bacillus* sp. YM-1 represents group I. DATAs of group II are colored grey. The residues coordinating the α-carboxylate group of substrates are highlighted in bold. Known second-shell residues are underlined.

TA	Amino Acid Motifs in the Active Site [13]		
BCAT from *Escherichia coli*	^31^YxxxxFxGxR^40^	^95^**Y**xR^97^	^107^MxV^109^
(*R*)-ATA from *Aspergillus fumigatus*	^53^HxxxxYxVxS^62^	^113^FxE^115^	^125^x**R**x^127^
DATA from *Bacillus* sp. YM-1	^26^Fxxxx**Y**xVxK^35^	^88^HxY^90^	^98^**R**x**H**^100^
DATA from *Aminobacterium colombiense*	^27^**R**xxxxFxTxS^36^	^86^MxR^88^	-
DATA from *Curtobacterium pusillum*	^46^**R**xxxxFxTxA^60^	^115^FxK^117^	-
DATA from *Blastococcus saxobsidence*	^34^**R**xxxxFxSxA^43^	^94^VxR^96^	-
DATA from *Haliscomenobacter hydrossis*	^28^**R**xxxxFxYxL^37^	^88^GxR^90^	-
DATA from *Desulfomonile tiedjei*	^37^**R**xxxxFxAxR^37^	^101^LxD^103^	-

**Table 2:** Kinetic parameters of the overall transamination reaction *D-alanine + α-ketoglutarate*, catalyzed by the WT, T199N, and T199Q variants in 50 mM K-phosphate buffer, pH 8.0, at 50 °C, and the T43E variant in 50 mM K-phosphate buffer, pH 6.5, at 40 °C, and the reaction *(R)-PEA + α-ketoglutarate*, catalyzed by WT DestiTA, in 50 mM Na-pyrophosphate buffer, pH 9.0, at 50 °C.

Substrate	Co-Substrate	Parameters			T_0.5_ ^1^, °C
		*k_cat_*, s^−1^	*K_m_*, mM	*k_cat_*/*K_m_*, s^−1^ M^−1^	
WT DestiTA					
D-alanine	α-ketoglutarate	5.8 ± 0.2	0.5 ± 0.1	11,600 ± 3000	55.0 ± 0.4 (64.4 ± 0.2)
α-ketoglutarate	D-alanine		0.11 ± 0.01	53,000 ± 8000	
(*R*)-PEA	α-ketoglutarate	0.7 ± 0.1	17 ± 2	70 ± 20	
α-ketoglutarate	(*R*)-PEA		0.36 ± 0.05	3300 ± 400	
The T43E variant					
D-alanine	α-ketoglutarate	0.15 ± 0.01	2.9 ± 0.8	52 ± 20	60.2 ± 0.3 (61.1 ± 0.4)
α-ketoglutarate	D-alanine		2.8 ± 0.9	54 ± 30	
The T199N variant					
D-alanine	α-ketoglutarate	5.0 ± 0.2	0.52 ± 0.07	10,000 ± 2000	54.7 ± 0.3 (63.2 ± 0.3)
α-ketoglutarate	D-alanine		0.21 ± 0.03	24,000 ± 4000	
The T199Q variant					
D-alanine	α-ketoglutarate	8.4 ± 0.4	50 ± 6	170 ± 30	50.9 ± 0.1 (65.7 ± 0.1)
α-ketoglutarate	D-alanine		0.45 ± 0.07	19,000 ± 4000	

^1^—Half-transition temperature (T_0.5_) of the thermal unfolding was measured in 50 mM K-phosphate buffer, pH 8.0, for 2 µM apoenzyme of the variants of DestiTA. The values in the brackets correspond to T_0.5_ of the variants in the presence of 500 µM PLP.

## Data Availability

The data presented in this study are available on request from the corresponding author.

## Supplementary Materials

The following supporting information can be downloaded at https://www.mdpi.com/article/10.3390/ijms26178536/s1.
