# Supplementary material for: Contribution of Second-Shell Residues to PLP-Dependent Transaminase Catalysis: A Case Study of D-Amino Acid Transaminase from Desulfomonile tiedjei
Source: Int J Mol Sci. 2025 Sep 2;26(17):8536. doi: 10.3390/ijms26178536 (PMC12429042; doi:10.3390/ijms26178536)
Supplement: Supplementary file 1 [file ijms-26-08536-s001.zip › ijms-3809256-supplementary.pdf]

Supplementary material

Contribution of second-shell residues to PLP-dependent transaminase catalysis: a case study of D-amino acid transaminase from *Desulfomonile tiedjei*

Alina K. Bakunova[1*†], Iuliia V. Rudina[1†], Vladimir O. Popov[12] and Ekaterina Yu. Bezsudnova[1]

[1]Bach Institute of Biochemistry, Research Centre of Biotechnology of the Russian Academy of Sciences, Moscow 119071, Russia;

[2]Department of Biology, Lomonosov Moscow State University, Moscow 119991, Russia.

* Corresponding author E-mail: a.bakunova@fbras.ru

[†] These authors contributed equally to this work.

Contents:

Table S1-S7 – pp. 2-9

Figure S1-S8 – pp. 10-15

Table S1. The specific activity of DestiTA in the overall transamination reactions between 5 mM amino donor and 2 mM amino acceptor in K-phosphate buffer, pH 8.0, at 40 °C.

**Amino Donor**	**Amino Acceptor**	**Specific Activity, U/mg**
D-alanine	α-ketoglutarate	2.8 ± 0.1
	4-methyl-2-oxovalerate	0.025 ± 0.005
L-alanine	α-ketoglutarate	ND
(*S*)-PEA		ND
(*R*)-PEA		0.28 ± 0.01
D-glutamate	pyruvate	5.0 ± 0.2
	3-methyl-2-oxobutyrate	0.84 ± 0.04
	phenylpyruvate	0.13 ± 0.01
	α-tetralone	ND
	indanone	ND
	acetophenone	ND
(*S*)-PEA	pyruvate	ND
(*R*)-PEA		0.46 ± 0.05

ND – not detected

Table S2. HPLC analysis condition.

**Conversion determination**
The α-keto acid concentrations in the aliquots were analyzed by Acta Purifier (Cytiva, Marlborough, MA, USA) equipped with a reverse-phase C18 column (Zorbax Eclipse XDB-C18, 5μm, 4.6×150 mm, (Agilent Technologies, Inc., Santa Clara, CA, USA)) equilibrated in 20 mM NaH_2PO_4, pH 2.2, 5% (*v*/*v*) methanol at 1.0 mL/min at 25 °C with detection at 210 nm. The retention time of 3-methyl-2-oxobutyrate was 6.1 min.
**Derivatization and HPLC conditions**
The chiral analysis of products of reactions was performed by HPLC using the reverse-phase C18 column with the UV detector set at 340 nm. Deproteinized samples were derivatized with Marfey's reagent (Sigma, St. Louis, MO, USA) according to Pavkov-Keller et al [1]. Briefly, 25 µl of Marfey's reagent (28 mM in acetonitrile) and 10 µl $NaHCO_3$ (1 M) were added to 10 µl sample and incubated at 50 °C for 2 h. The reaction mixture was cooled, and then the reaction was stopped by adding 3 µl of 4 M HCl and 10 µl of 100% methanol. The retention time of L- and D-valine were 16.5 and 19.4 min, respectively.

Instrument:	ÄKTA Purifier, Cytiva, Marlborough, MA, USA
Column:	Zorbax Eclipse XDB-C18, 5 µM, 4.6 × 150 mm, Agilent Technologies, Inc., Santa Clara, CA, USA
Buffer A:	0.1% trifluoroacetic acid in water
Buffer B:	0.1% trifluoroacetic acid in 100% methanol
Elution:	linear gradient of Buffer B from 20% to 70% in 15 min.
Flow rate:	1.0 ml/min
Temperature:	25 °C
Injection volume:	10 µL
Detection:	UV, 340 nm

Chromatograms of standards of D- and L-valine at concentration of 50 mM (red) and sample (black) derivatized with Marfey's reagent (MR).

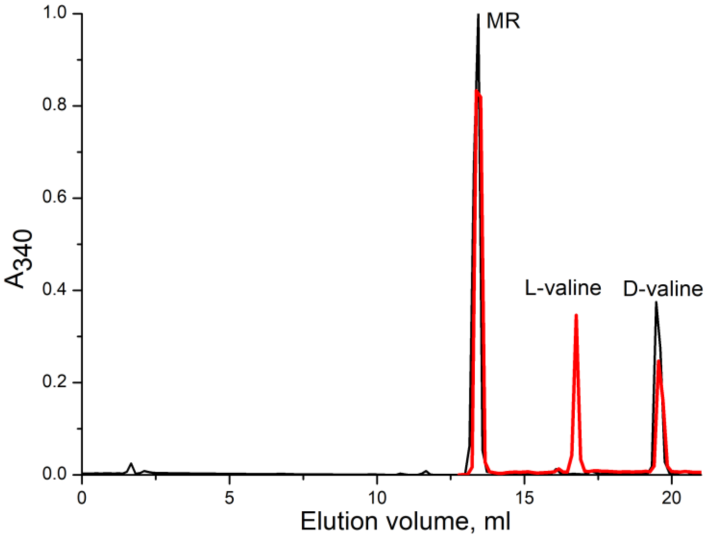

Table S3. The length of the bond between the side chains of the glutamate residue coordinating the N1 atom of PLP and threonine (E/OE1–T/OG1 bond) or asparagine residue (E/OE1–N/ND2 bond) in the active sites of IV fold type TAs, observed in an independent crystallographic unit (the range is presented when there are several non-identical subunits).

PDB ID	Length of E/OE1–T/OG1 bond, Å
**DATA**	
8AHR	4.5
8PNW	5.1
6Q1R	4.8-5.0
	**Length of E/OE1–N/ND2 bond, Å**
**DATA**	
4DAA	3.5-3.4
4TM5	3.1
7P7X	3.2
5K3W	3.8-3.9
**BCAT**	
5E25	3.9-4.1
5MQZ	3.9
1I1K	4.2
6NST	4.2-4.5
1WRV	3.9
4WHX	3.3-3.8
5CE8	4.0
4TVI	3.7-3.8
3CSW	3.5-3.6
4JXU	3.5
3UZB	3.5
6JIF	3.1-3.4
**(*R*)-ATA**	
3WWH	3.4
6SNL	3.3-3.4
6XU3	3.5-3.6
6XWB	3.5
4CE5	3.6
6FTE	3.6
4CHI	3.6
4CMD	3.5

Table S4. The dependence of the specific activity of the T43E apoenzyme on the PLP concentration in the reaction mixture. The reaction condition was 25 mM D-alanine, 10 mM α-ketoglutarate, 0.05 mg/ml T43E variant in 50 mM K-phosphate buffer, pH 6.5, at 40 °C.

[PLP], μM	Specific activity, U/mg
30	0.160 ± 0.006
100	0.173 ± 0.007
300	0.168 ± 0.004
600	0.168 ± 0.008

Table S5. Sequences of primers used for mutagenesis, the mutations are underlined.

T43E.R; 5’-GTGCGGCAAGCCTCGAAGATACGGTAACC-3’<br>CheckT43E.F; 5’-GGTTACCGTATCTTCGAG-3’
T199N.F; 5’-CTCTACCTTCAACGTCTTCTTCGTAAACGC-3’<br>CheckT199N.R; 5’-GCGTTTACGAAGAAGACGT-3’
T199Q.F; 5’-CTCTACCTTCCAAGTCTTCTTCGTAAACGC-3’<br>CheckT199Q.R; 5’-GCGTTTACGAAGAAGACTTG-3’

Table S6. Steady-state kinetics of the transamination reactions catalyzed by the DestiTA variants.

The transamination reaction between D-alanine and α-ketoglutarate catalyzed by WT DestiTA

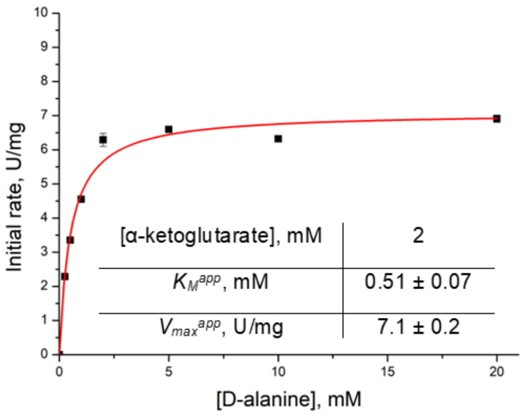

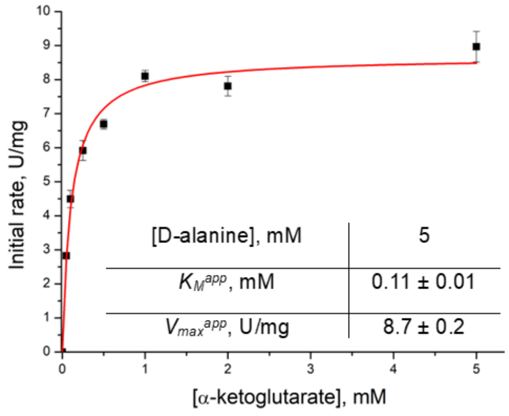

The transamination reaction between D-alanine and α-ketoglutarate catalyzed by the T43E variant

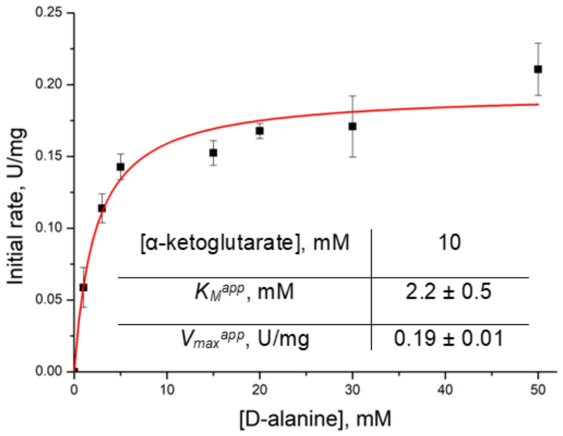

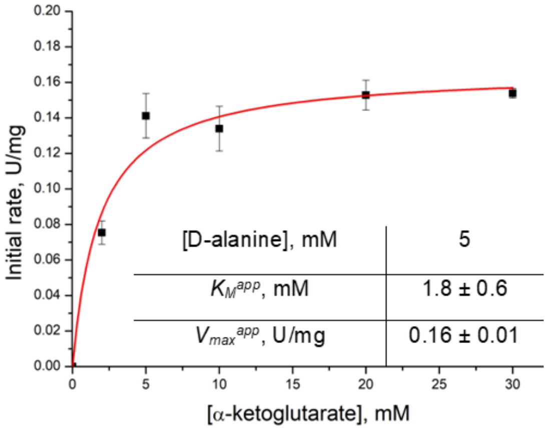

The transamination reaction between D-alanine and α-ketoglutarate catalyzed by the T199N variant

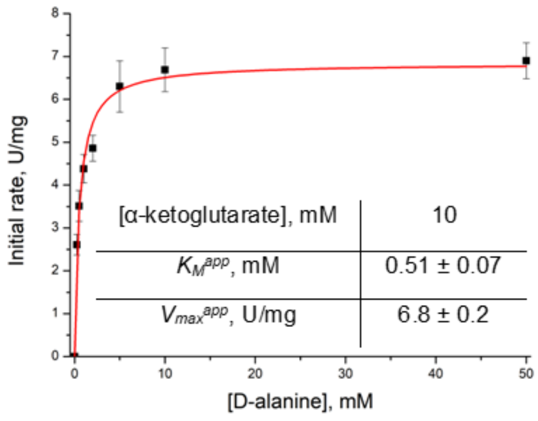

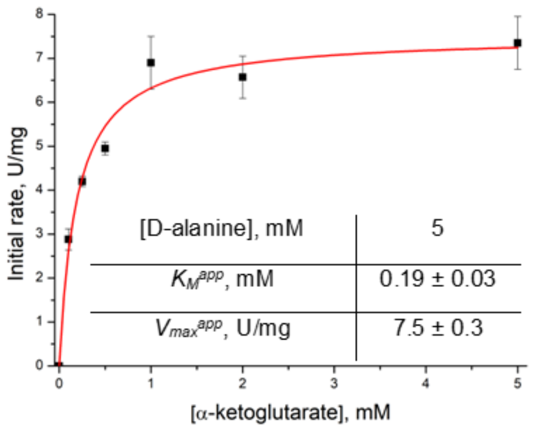

The transamination reaction between D-alanine and α-ketoglutarate catalyzed by the T199Q variant

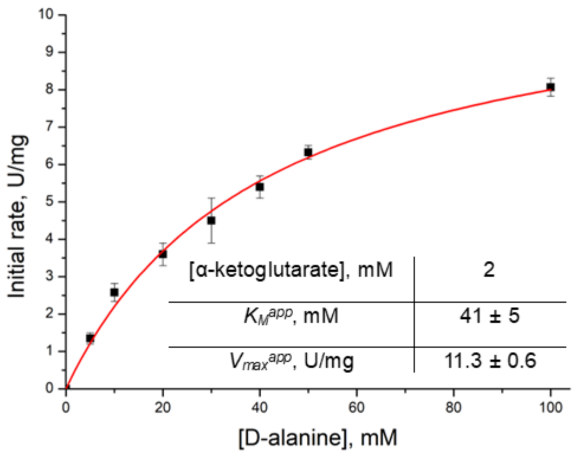

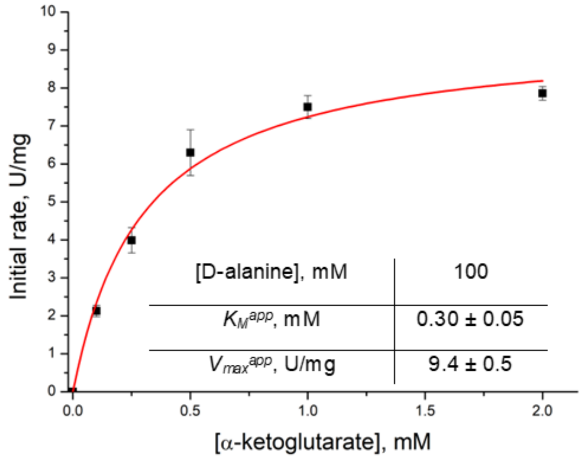

The transamination reaction between (*R*)-PEA and α-ketoglutarate catalyzed by WT DestiTA

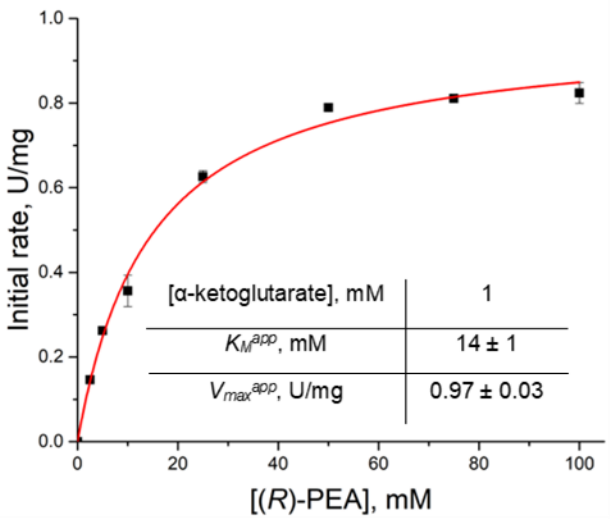

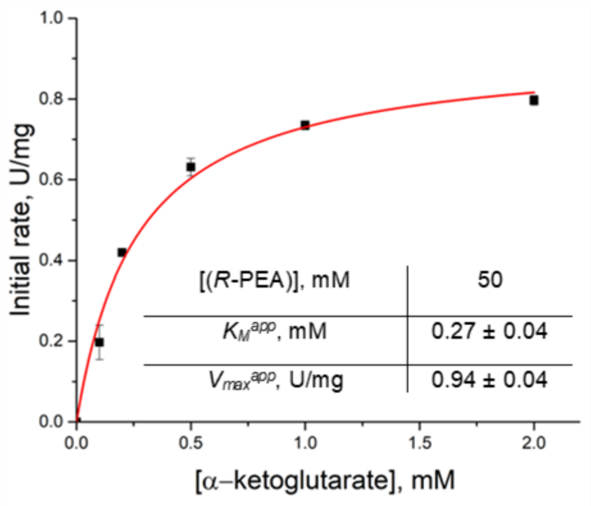

Table S7. Topology for the PLP molecule.

The PLP molecule, bonded to lysine residue

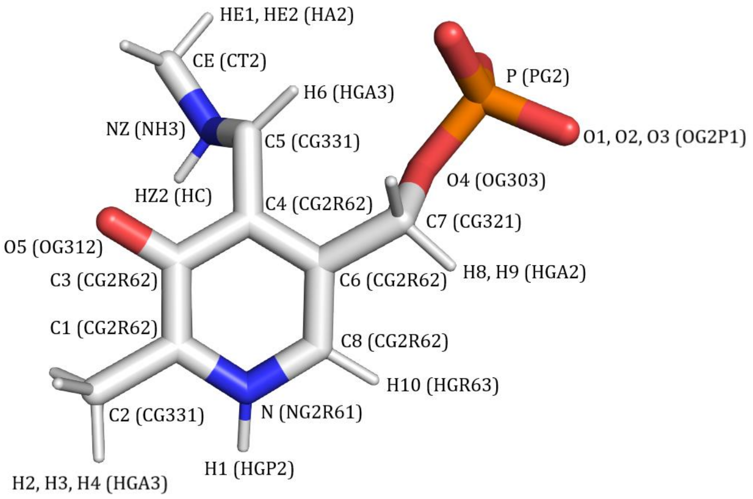

Topology file for PLP:

```
RESI plp           -2.000 ! param penalty=  57.500 ; charge penalty=
33.961
GROUP                   ! CHARGE   CH_PENALTY
ATOM P      PG2      1.103 !   2.500
ATOM O1     OG2P1   -0.900 !   0.000
ATOM O2     OG2P1   -0.900 !   0.000
ATOM O3     OG2P1   -0.900 !   0.000
ATOM O4     OG303   -0.438 !  12.030
ATOM N      NG2R61  -0.413 !  30.628
ATOM C1     CG2R62  -0.101 !  33.961
ATOM C2     CG331   -0.205 !  22.664
ATOM C3     CG2R62   0.553 !  26.775
ATOM O5     OG312   -0.693 !  21.664
ATOM C4     CG2R62  -0.452 !  19.611
ATOM C5     CG331   -0.202 !  10.914
ATOM C6     CG2R62   0.070 !  15.808
ATOM C7     CG321   -0.138 !  11.291
ATOM C8     CG2R62   0.255 !   9.896
ATOM H1     HGP2     0.422 !   2.500
ATOM H2     HGA3     0.090 !   1.350
ATOM H3     HGA3     0.090 !   1.350
ATOM H4     HGA3     0.090 !   1.350
ATOM H5     HGA3     0.090 !   0.000
ATOM H6     HGA3     0.090 !   0.000
ATOM H7     HGA3     0.090 !   0.000
ATOM H8     HGA2     0.090 !   0.000
ATOM H9     HGA2     0.090 !   0.000
ATOM H10    HGR63    0.219 !   0.517

BOND P    O1
BOND P    O3
BOND O2   P
BOND O4   P
BOND N    C8
BOND N    H1
BOND C1   N
```

```
BOND C2   H3
BOND C1   C2
BOND C2   H2
BOND C3   C4
BOND C3   C1
BOND O5   C3
BOND C4   C6
BOND C5   H5
BOND C5   C4
BOND C6   C7
BOND C6   C8
BOND C7   O4
BOND C7   H9
BOND C8   H10
BOND H4   C2
BOND H6   C5
BOND H7   C5
BOND H8   C7
```

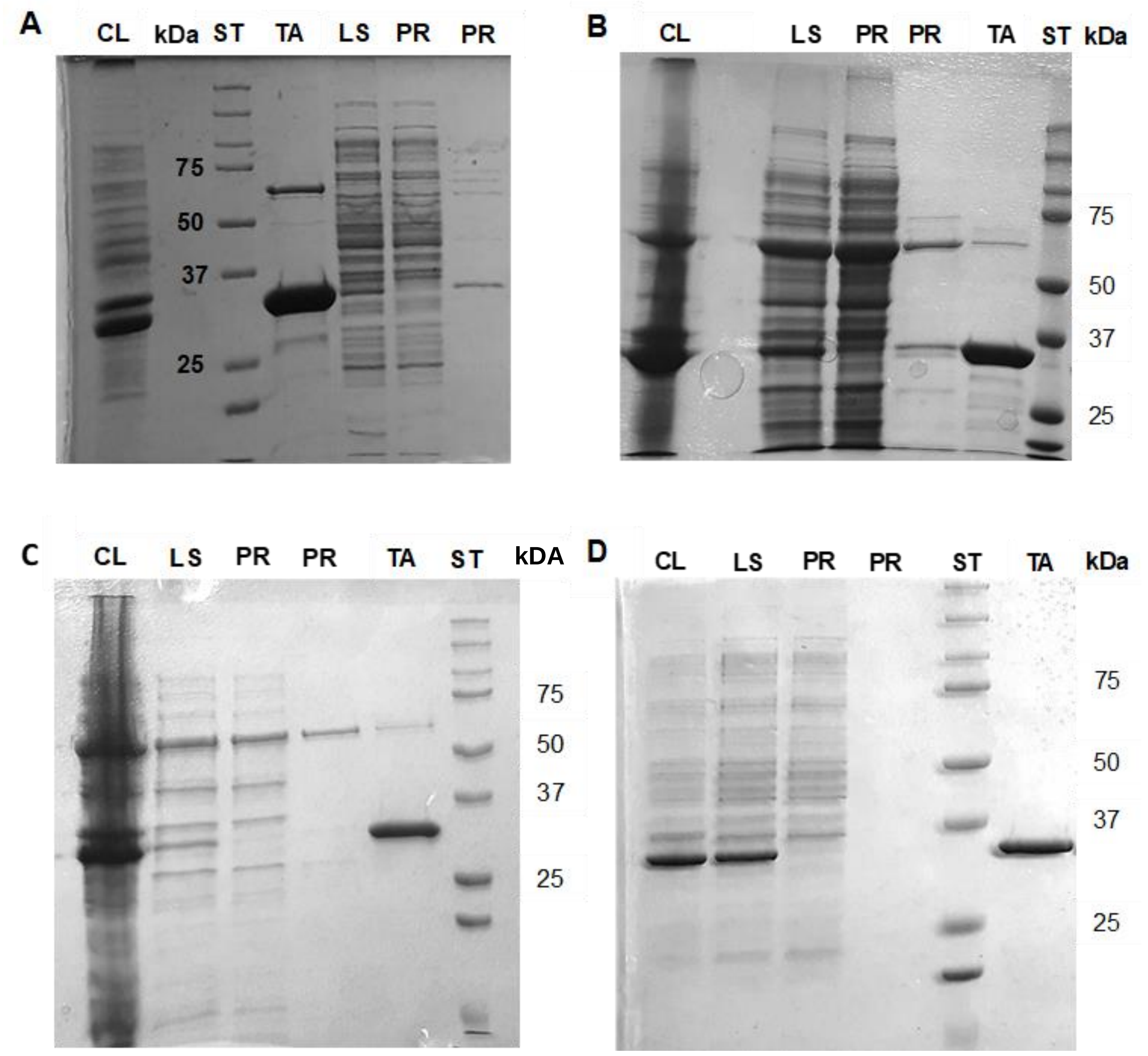

Figure S1. SDS-PAGE of stage expression and purification of DestiTA. **A** – WT, **B** – T199N, **C** – T199Q, **D** – T43E. CL, *E.coli* cell after IPTG induction; LS, cell free lysate obtained by sonication, centrifugation and filtration; PR, fractions of purification stages without protein of interest; TA, fraction of DestiTA after HisTrap HP column; ST, Precision Plus Protein Dual Color Standards (Bio-Rad Laboratories, USA).

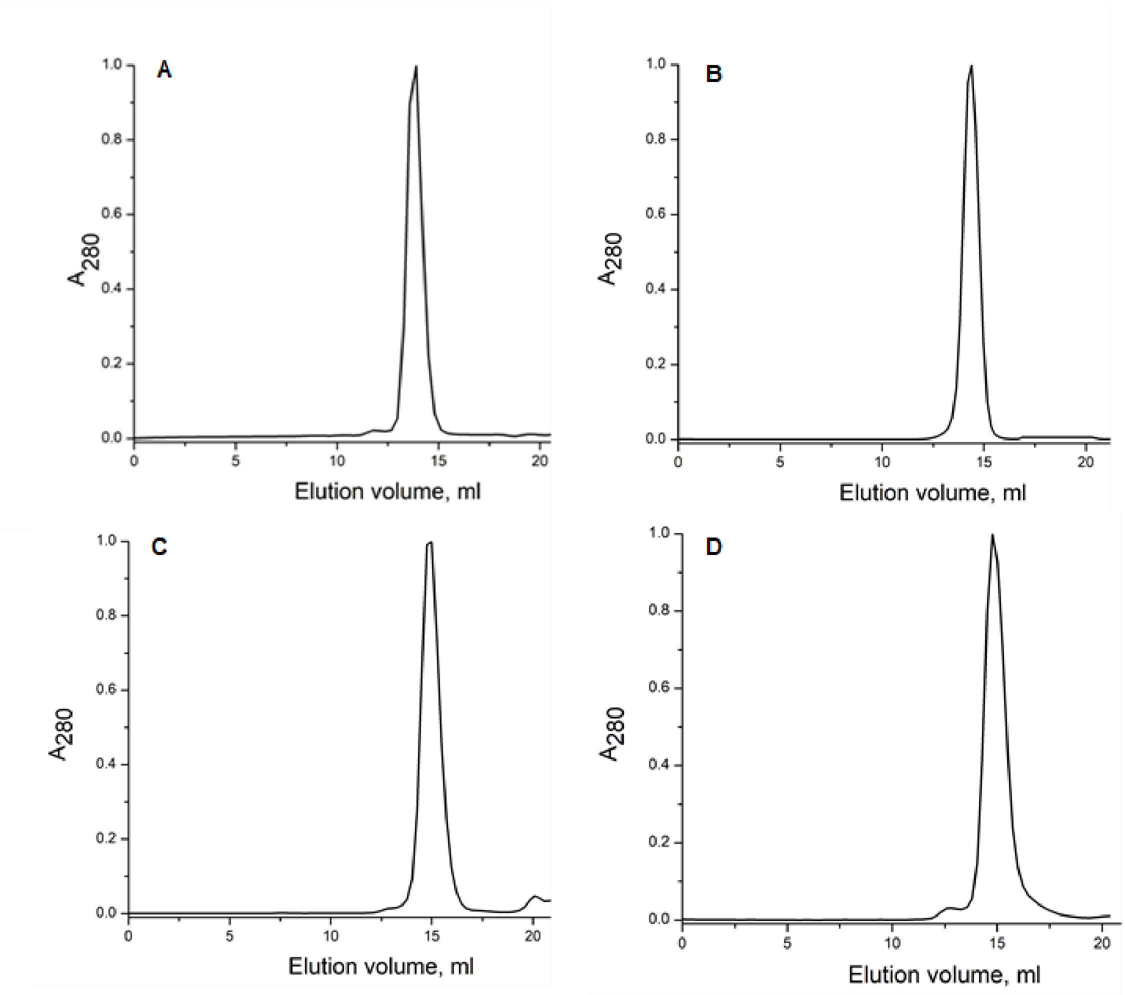

Figure S2. The gel filtration elution profile for the DestiTA variants. The major peak corresponds to the dimer of the WT (A), T43E (B), T199N (C), and T199Q (D) DestiTA variants.

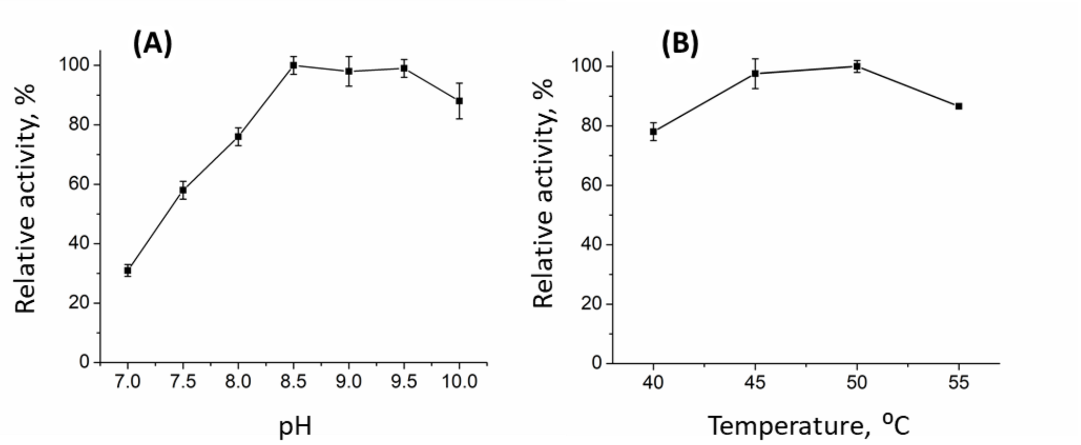

Figure S3. The pH (**A**) and temperature (**B**) dependences of the activity of the WT DestiTA in the overall transamination reaction *(R)-PEA + α-ketoglutarate*; 100% corresponds to 0.39 ± 0.01 U/mg (A), 0.41 ± 0.01 U/mg (B).

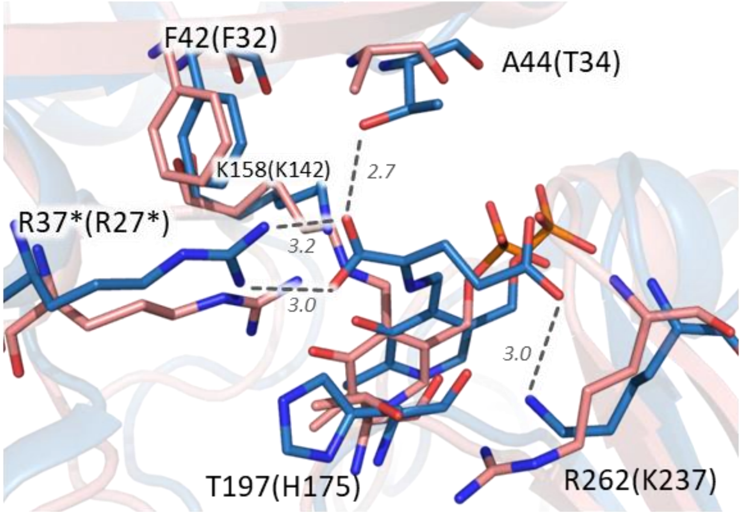

Figure S4. The superposition of the active site of WT DestiTA (pink) and AmicoTA (blue, PDB ID 8AYK).

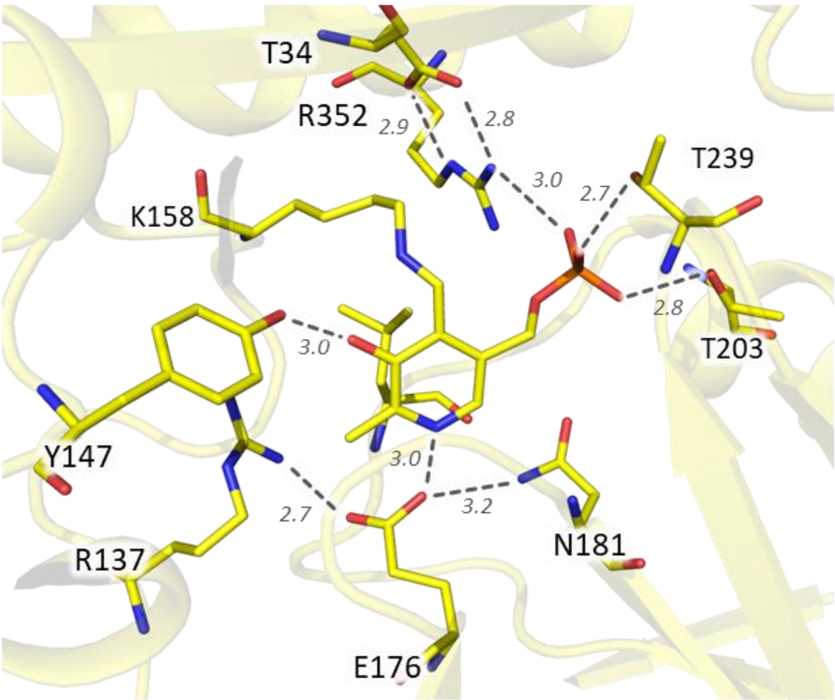

Figure S5. Binding PLP in the active site of Halhy (PDB ID: 7P7X).

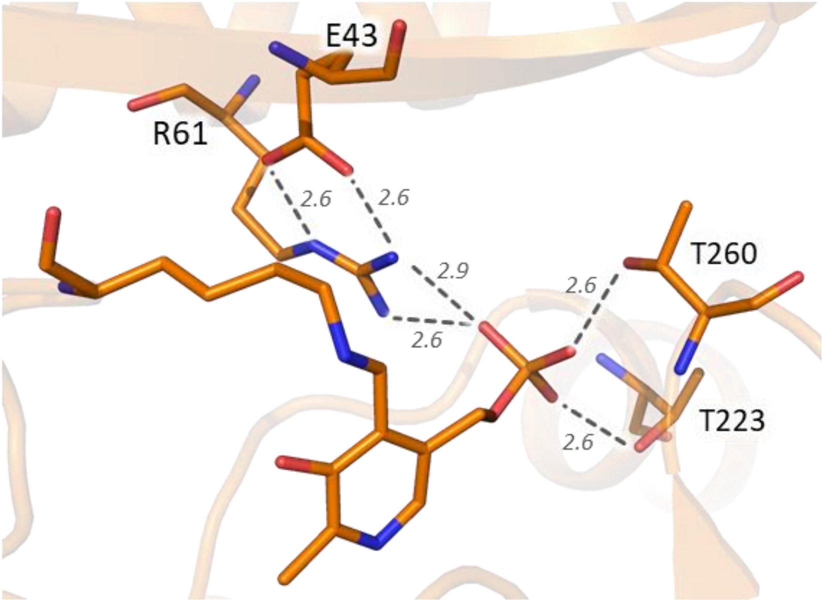

Figure S6. The active site of the T43E DestiTA variant.

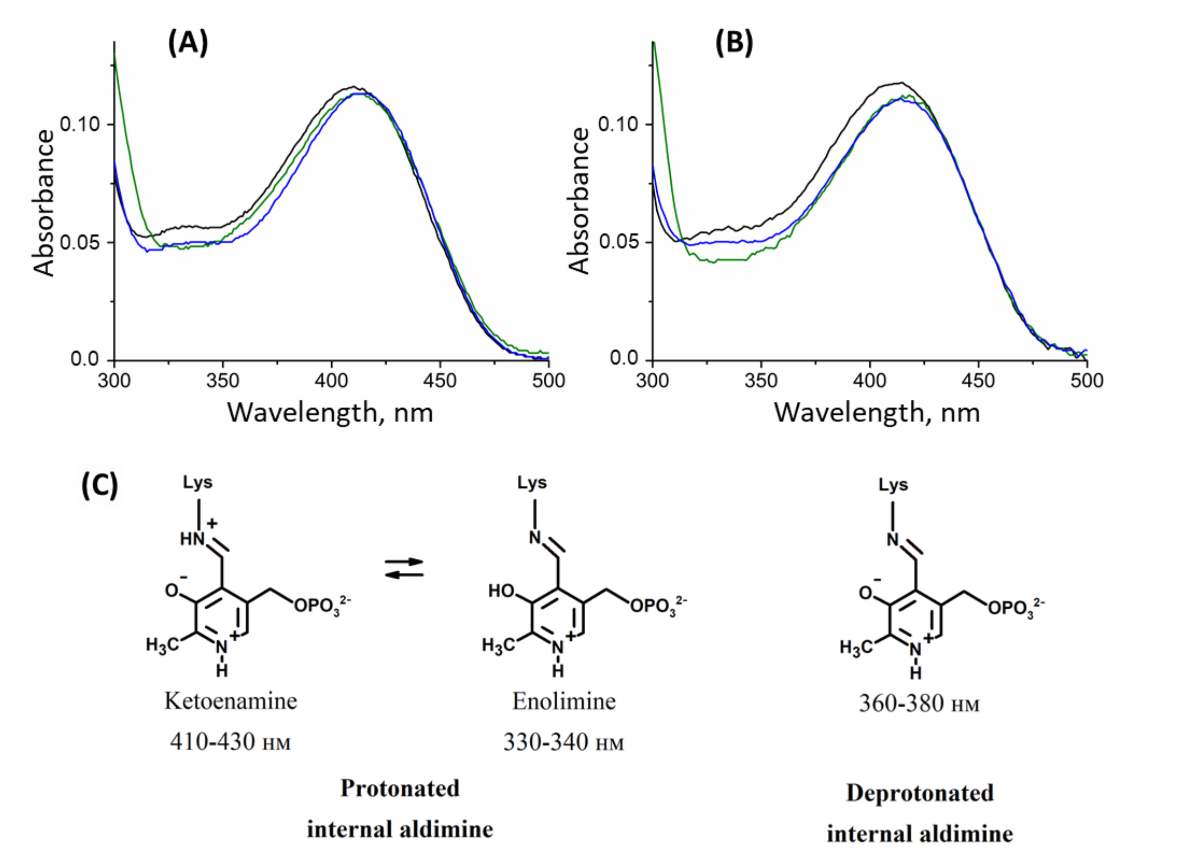

Figure S7. The absorption spectra of 14 μM PLP form of the WT (A) and the T199Q variant (B) in 50 mM K-phosphate buffer, pH 8.0 (black), Na-pyrophosphate buffer, pH 9.0 (blue) and pH 10.0 (green) at 25 °C. (C) The structurs of protonated and deprotonated internal aldimine with the indicated absorption maxima.

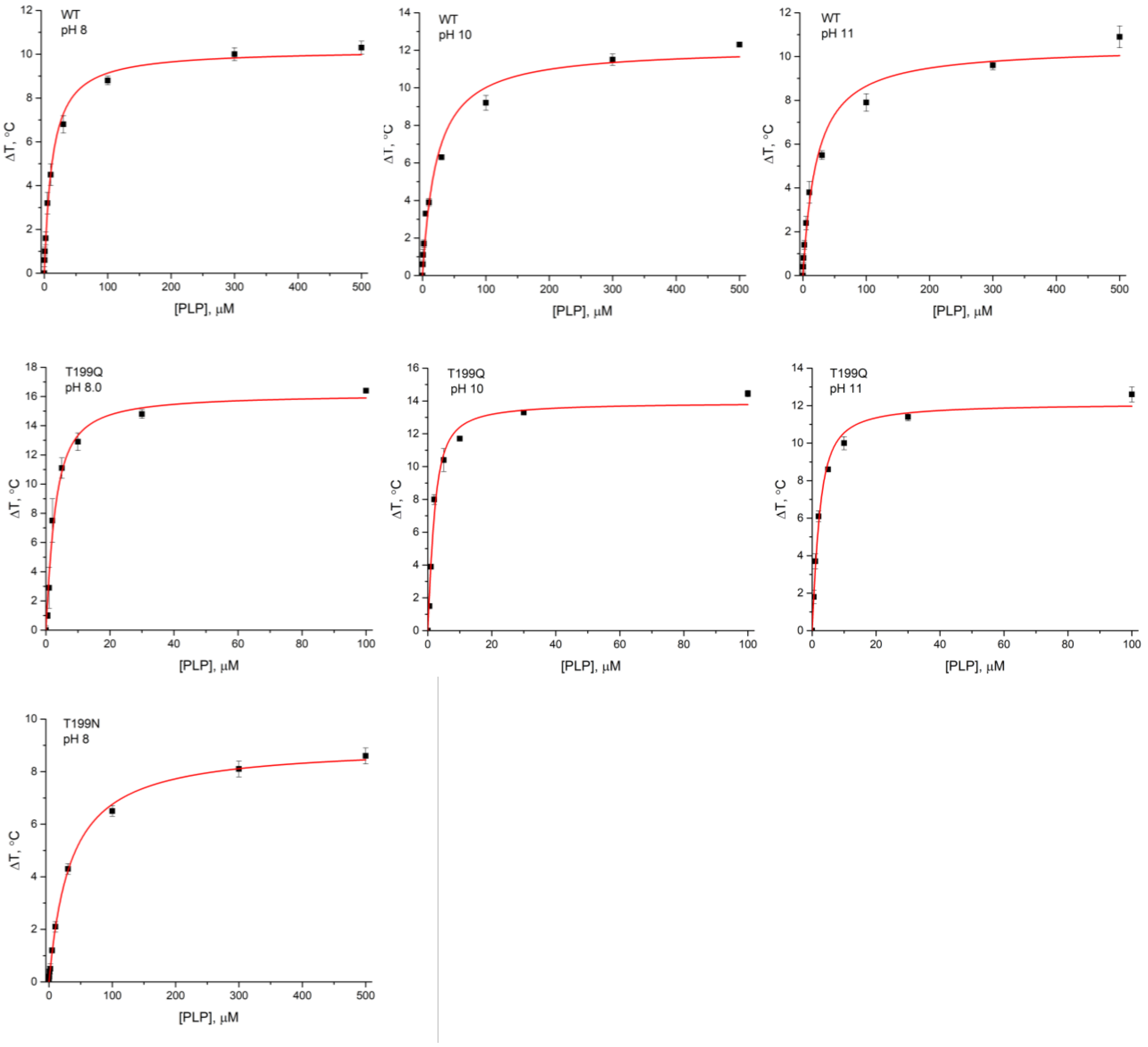

Figure S8. The dependence of the midpoint temperature shift of the thermal denaturation of 2 μM apoenzymes on the PLP added in the concentration range 0-500 mkM.

References

1. Pavkov-Keller, T.; Strohmeier, G.A.; Diepold, M.; Peeters, W.; Smeets, N.; Schürmann, M.; Gruber, K.; Schwab, H.; Steiner, K. Discovery and structural characterisation of new fold type IV-transaminases exemplify the diversity of this enzyme fold. *Sci. Rep.* **2016**, *6*, 38183, doi:10.1038/srep38183.
